# The Investigation of Serum Vaspin Level in Atherosclerotic Coronary Artery Disease

**DOI:** 10.4021/jocmr841w

**Published:** 2012-03-23

**Authors:** Mehmet Ali Kobat, Ahmet Celik, Mehmet Balin, Yakup Altas, Adil Baydas, Musa Bulut, Suleyman Aydin, Necati Dagli, Mustafa Ferzeyn Yavuzkir, Selcuk Ilhan

**Affiliations:** a Department of Cardiology, Elazig Education and Research Hospital, Elazig, Turkey; b Department of Cardiology, Firat University Medical Faculty, Elazig, Turkey; c Department of Biochemistry, Firat University Medical Faculty, Elazig, Turkey; d Department of Pharmacology, Firat University Medical Faculty, Elazig, Turkey

## Abstract

**Background:**

It was speculated that fatty tissue originated adipocytokines may play role in pathogenesis of atherosclerosis. These adipocytokines may alter vascular homeostasis by effecting endothelial cells, arterial smooth muscle cells and macrophages. Vaspin is a newly described member of adipocytokines family. We aimed to investigate whether plasma vaspin level has any predictive value in coronary artery disease (CAD).

**Methods:**

Forty patients who have at least single vessel ≥ 70 % stenosis demostrated angiographically and 40 subjects with normal coronary anatomy were included to the study. The vaspin levels were measured from serum that is obtained by centrifigation of blood and stored at -20 ^o^C by ELISA method. The length, weight and body mass index of patients were measured. Biochemical parameters including total cholesterol, low density lipoprotein, high density lipoprotein, creatinine, sodium, potassium, hemoglobine, uric acid and fasting glucose were also measured.

**Results:**

Biochemical markers levels were similar in both groups. Serum vaspin levels were significantly lower in CAD patients than control group (respectively; 256 ± 219 pg/ml vs. 472 ( 564 pg/ml, P < 0.02). Beside this serum vaspin level was lower in control group with high systolic blood pressure.

**Conclusion:**

Serum vaspin levels were found significantly lower in patients with CAD than age-matched subjects with normal coronary anatomy. Vaspin may be used as a predictor of CAD.

**Keywords:**

Coronary artery disease; Vaspin; Adipokine

## Introduction

Inflammation was thought to be one of the major causes of early atherosclerosis and its’ progressive complications such as plaque rupture [1, 2]. Inflammation and blood flow are alternative pathogenic factors for plaque development [3, 4].

Recently, it was shown that adipokines including visseral adipose tissue-derived serine protease inhibitor (VASPIN) which have endocrine and local roles in atherosclerosis development are syntesized by adipose tissue [5, 6]. Adipose tissue derived factors including adipokines has been suggested as actors for premature and accelerated atherosclerosis in obese people [7]. VASPIN is a member of serine protease inhibitor family which has a regulator role in glukose and lipid metabolism [8]. The high blood vaspin concentration in obese subjects was shown [9] and it was also demonstrated in both non-obese and obese type 2 diabetic patients [10]. The relationship between VASPIN levels and coronary artery disease (CAD) was unclear in the literature.

In the present study we aimed to show the relationship between serum VASPIN levels and patients with angiographically proven coronary artery disease.

## Methods

In this study, we screened 40 consecutive patients with stable angina pectoris who had at least 70% stenosis in any coronary artery diagnosed by coronary angiography (CAD Group) and 40 subjects with normal coronary anatomy that shown by coronary angiography (Control Group). Exclusion criteria consisted acute coronary syndrome, morbid obesity, history of diabetes mellitus and coronary artery disease, patients who had any revascularization theraphy, history of early menopause, heart failure and cardiomyopathy. The study was approved by the ethical review board of Firat University. All patients were informed about the study, and their written consent forms were obtained.

Blood samples were obtained within 24 hours of presentation. Blood samples for vaspin (Human Vaspin ELISA Kit, ALPCO IMMUNOASSAYS, Catalog Number: 44-VASHU-E01)) were obtained and centrifuged then stored at -20 ^o^C. Serum vaspin levels were analysed after the blood samples of all study patients were obtained. BMI was calculated as weight divided by heighy squared (kg/m^2^) according to the World Health Organization [11].

A conventional coronary angiography was performed with Philips Integris 5000 equipment (Philips Medical Systems, Best, Netherlands) in patients with stable angina pectoris. After obtaining images by standard approaches, each angiogram was interpreted by two independent cardiologists. Eighty patients were divided into two groups according to the angiographic properties of their coronary angiographies. The CAD group consisted of 40 patients with at least 70% stenosis in any coronary artery the control group consisted of 40 patients with normal coronary anatomy.

Continuous variables were given as mean ± SD; categorical variables were defined as a percentage. A value of P < 0.05 was considered to be significant. Comparisons between the groups were carried out using an independent- samples t-test. Correlation analyses were performed using the Pearson coefficient of correlation. SPSS 15.0 software was used for basic statistical analysis (Version 15, SPSS Inc., Chicago, IL, USA).

## Results

The demographic properties of patients in two groups were summarized in Table 1. There were no significant difference between groups in age, gender, systolic blood pressure, diastolic blood pressure, body mass index, history of hypertension, family history, hyperlipidemia and smoke (Table 1).

**Table 1:** The Demographic Properties of Two Groups

	CAD Group (n = 40)	Control Group (n = 40)	P value
Age (years)	60 ± 11	56 ± 8	0.1
Female (%)	33	43	0.2
Hypertension (%)	48	53	0.4
Hyperlipidemia (%)	55	50	0.4
Smoke (%)	35	23	0.1
Body Mass Index (kg/m^2^)	26.7 ± 3.6	27 ± 5.4	0.5
Systolic Blood Pressure (mmHg)	122 ± 14	123 ± 15	0.6
Diastolic Blood Pressure (mmHg)	76 ± 8	76 ± 9	0.8
Family History (%)	45	38	0.3

Data expressed as mean ± SD or percentage. P < 0.05 was accepted statistically significant.

The biochemical properties of the study patients were seen in Table 2. There were no difference between the groups in fasting glucose, hemoglobin, lipid profile, creatinin, sodium, potassium and uric acid levels (Table 2). The serum VASPIN levels were significantly lower in CAD group than control group (256 ± 219 pg/mL and 472 ± 564 pg/mL, respectively, P = 0.02).

**Table 2:** The Biochemical Properties of Two Groups

	CAD Group (n = 40)	Control Group (n = 40)	P value
Fasting glucose (mg/dL)	99 ± 23	98 ± 17	0.7
Hemoglobin (g/dL)	13.4 ± 1.2	13.6 ± 3.5	0.7
Creatinin (mg/dL)	1.1 ± 1.0	1.1± 1.3	0.9
Sodyum (mmol/L)	140 ± 4	140 ± 4	0.6
Potasyum (meq/L)	4.3 ± 0.3	4.3 ± 0.3	0.4
Uric acid (mg/dL)	5.6 ± 1.2	6.0 ± 1.7	0.2
Lipid Profile (mg/dL)			
LDL-C	123 ± 28	122 ± 26	0.9
HDL-C	41 ± 4	43 ± 6	0.1
Total-C	195 ± 40	193 ± 32	0.6
Vaspin (pg/mL)	256 ± 219	472 ± 564	0.02

Data expressed as mean± SD. P < 0.05 was accepted statistically significant. LDL-C: low density lipoprotein cholesterol, HDL-C: high density lipoprotein cholesterol, Total-C: total cholesterole.

There were statistically significant negative correlation between serum VASPIN levels and systolic blood pressure in control group patients (r:-0.349, P = 0.02). VASPIN levels were weakly negative correlated with diastolic blood pressure in control group (r = -0.299, P = 0.06).

## Discussion

This is the first study demonstrating low serum VASPIN levels in coronary artery disease comparing to the age-matched people with normal coronary anatomy. We excluded the subjects with morbid obesity, diabetes mellitus, metabolic syndrome, heart failure, previous CAD history which might effect the VASPIN levels. According to our study we thought that VASPIN may have a protective role for coronary artery disease in people.

There were many studies aimed to prevent CAD because of the mortal complications of CAD and high cost of treatment. Even though it was known that inflammation has an important role in the both beginning and progression of atherosclerotic disease, the biochemical and cellular mechanisms are not clear [12]. The trigger of inflammation is not yet understood. In recent years it was shown that adipose tissue synthesis many bioactive substances participating into the circulation such as adiponectine [5], leptine [13], tumor necrosis factor-alfa [14], plasminogen activator inhibitor-1 [15], interleukine-6 [16], resistine [17], and various growth factors named adipocines. These substances have endocrine and local effects in atherosclerosis [18]. VASPIN is a member of serine protease inhibitor family that have a regulatuar role in glucose and lipid metabolism and its’ expression in visseral adipose tissue in the peak concentration of insulin and obesity in Otsuka Long-Evans Tokushima Fatty (OLETF) rats was shown [8]. But its’ physiological role is stil unknown.

Kadoglou et al. showed the lower vaspin levels in CAD compared with healty subjects [19], but in the present study we showed the lower VASPIN levels in CAD compared with age-matched subjects who had same symptoms with angiographically normal coronary anatomy. Austet et al. could not found any relation between serum VASPIN levels and carotid artery stenosis but they demonstrated that lower serum VASPIN levels had correlation with recent ischemic events in patients with carotid artery stenosis [17]. So according to these studies, we think that the low serum VASPIN levels may be a predictor of atherosclerotic disease.

### Limitations

Large and long-term follow-up studies are needed. The small number of patients was the main limitation of the present study. Many risk factors of CAD such as diabetes mellitus, obesity might affect the VASPIN levels, so it was difficult to use it in all patients in the diagnosis of CAD.

### Conclusion

We showed the lower serum VASPIN levels in patients with CAD comparing to the gender and age-matched subjects with angiographically normal coronary anatomy. So low VASPIN levels may be an indicator of CAD.

## References

[1] Ross R (1993). The pathogenesis of atherosclerosis: a perspective for the 1990s. Nature.

[2] Hansson GK (1993). Immune and inflammatory mechanisms in the development of atherosclerosis. Br Heart J.

[3] Libby P (2002). Inflammation in atherosclerosis. Nature.

[4] Cunningham KS, Gotlieb AI (2005). The role of shear stress in the pathogenesis of atherosclerosis. Lab Invest.

[5] Berg AH, Combs TP, Scherer PE (2002). ACRP30/adiponectin: an adipokine regulating glucose and lipid metabolism. Trends Endocrinol Metab.

[6] Ahima RS, Osei SY (2008). Adipokines in obesity. Front Horm Res.

[7] Fantuzzi G, Mazzone T (2007). Adipose tissue and atherosclerosis: exploring the connection. Arterioscler Thromb Vasc Biol.

[8] Hida K, Wada J, Eguchi J, Zhang H, Baba M, Seida A, Hashimoto I (2005). Visceral adipose tissue-derived serine protease inhibitor: a unique insulin-sensitizing adipocytokine in obesity. Proc Natl Acad Sci U S A.

[9] Youn BS, Kloting N, Kratzsch J, Lee N, Park JW, Song ES, Ruschke K (2008). Serum vaspin concentrations in human obesity and type 2 diabetes. Diabetes.

[10] El-Mesallamy HO, Kassem DH, El-Demerdash E, Amin AI (2011). Vaspin and visfatin/Nampt are interesting interrelated adipokines playing a role in the pathogenesis of type 2 diabetes mellitus. Metabolism.

[11] (2000). Obesity: preventing and managing the global epidemic. Report of a WHO consultation. World Health Organ. Tech Rep Ser.

[12] Ross R (1999). Atherosclerosis--an inflammatory disease. N Engl J Med.

[13] Van Harmelen, Reynisdottir S, Eriksson P, Thorne A, Hoffstedt J, Lonnqvist F, Arner P (1998). Leptin secretion from subcutaneous and visceral adipose tissue in women. Diabetes.

[14] Moller DE (2000). Potential role of TNF-alpha in the pathogenesis of insulin resistance and type 2 diabetes. Trends Endocrinol Metab.

[15] Alessi MC, Peiretti F, Morange P, Henry M, Nalbone G, Juhan-Vague I (1997). Production of plasminogen activator inhibitor 1 by human adipose tissue: possible link between visceral fat accumulation and vascular disease. Diabetes.

[16] Fried SK, Bunkin DA, Greenberg AS (1998). Omental and subcutaneous adipose tissues of obese subjects release interleukin-6: depot difference and regulation by glucocorticoid. J Clin Endocrinol Metab.

[17] Steppan CM, Bailey ST, Bhat S, Brown EJ, Banerjee RR, Wright CM, Patel HR (2001). The hormone resistin links obesity to diabetes. Nature.

[18] Aust G, Richter O, Rohm S, Kerner C, Hauss J, Kloting N, Ruschke K (2009). Vaspin serum concentrations in patients with carotid stenosis. Atherosclerosis.

[19] Kadoglou NP, Gkontopoulos A, Kapelouzou A, Fotiadis G, Theofilogiannakos EK, Kottas G, Lampropoulos S (2011). Serum levels of vaspin and visfatin in patients with coronary artery disease-Kozani study. Clin Chim Acta.

